# Supplementary material for: Actions of Esomeprazole on the Maternal Vasculature in Lean and Obese Pregnant Mice with Impaired Nitric Oxide Synthesis: A Model of Preeclampsia
Source: Int J Mol Sci. 2022 Jul 25;23(15):8185. doi: 10.3390/ijms23158185 (PMC9330120; doi:10.3390/ijms23158185)
Supplement: Supplementary file 1 [file ijms-23-08185-s001.zip › ijms-1826306-supplementary.pdf]

Supplementary Materials

Actions of esomeprazole on the maternal vasculature in normal and obese pregnant mice with impaired nitric oxide synthesis: a model of preeclampsia

Natasha de Alwis, Natalie K Binder, Yeukai TM Mangwiro, Sally Beard, Natasha Pritchard, Elif Kadife, Bianca R Fato, Emerson Keenan, Fiona C Brownfoot, Tu'uhevaha J Kaitu'u-Lino, Natalie J Hannan

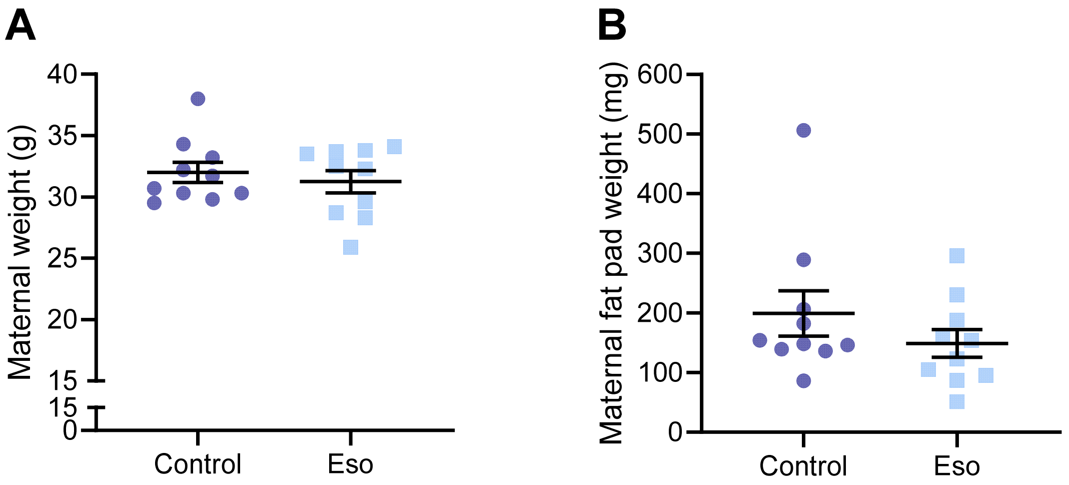

Supplementary Figure S1. Effect of esomeprazole treatment on the maternal body and fat pad weight of lean L-NAME mice. Esomeprazole treatment did not alter either body weight or fat pad weight. Data presented as mean ± SEM; n=10 mice/group.

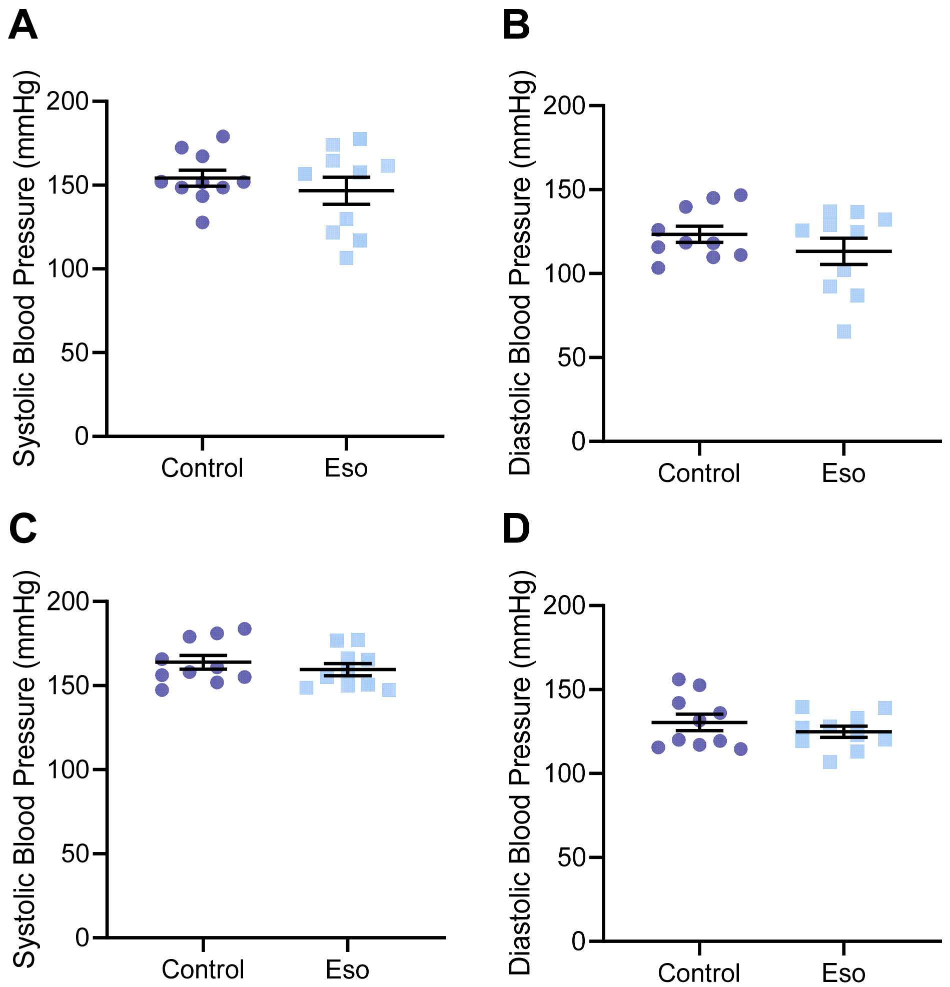

Supplementary Figure S2. Systolic and diastolic blood pressure of lean L-NAME mice treated with esomeprazole. Blood pressure was measured via tail cuff plethysmography at E14.5 (A, B) and E17.5 (C, D) of pregnancy. Esomeprazole treatment did not significantly alter systolic or diastolic blood pressure at either time point in these L-NAME mice. Results presented as mean ± SEM. n=10 mice/group.

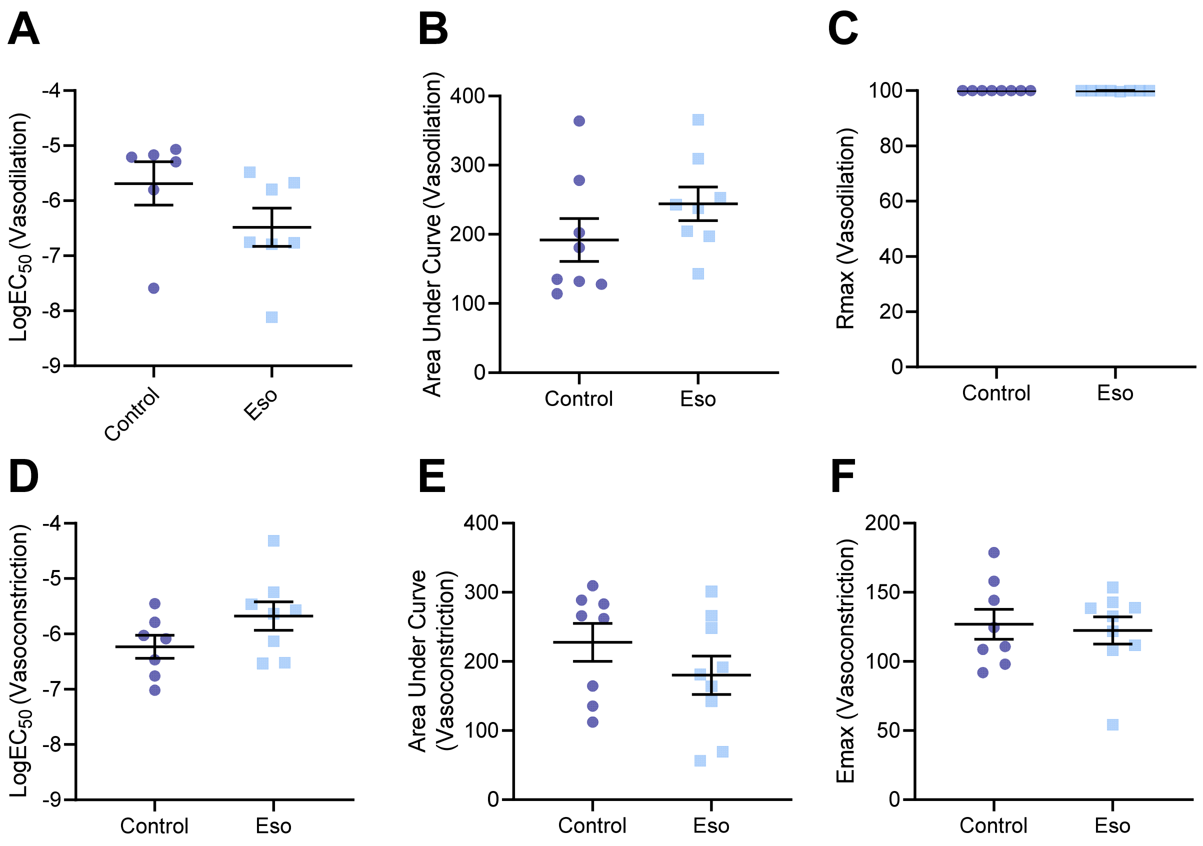

Supplementary Figure S3. Effect of esomeprazole on mesenteric arteries collected from lean L-NAME mice – corresponding parameters to myograph results in Figure 2. A) Vasodilation LogEC50, B) vasodilation area under the curve, and C) maximum relaxation (Rmax) to acetylcholine. D) Vasoconstriction LogEC50, E) vasoconstriction area under the curve, and F) maximum constriction (Emax) to phenylephrine. None of the parameters examined were significantly different in the response of the arteries collected from the L-NAME mice administered esomeprazole versus the controls. n=8 mice/group. LogEC50 from n=1 eso and n=2 control arteries was ambiguous and hence, excluded.

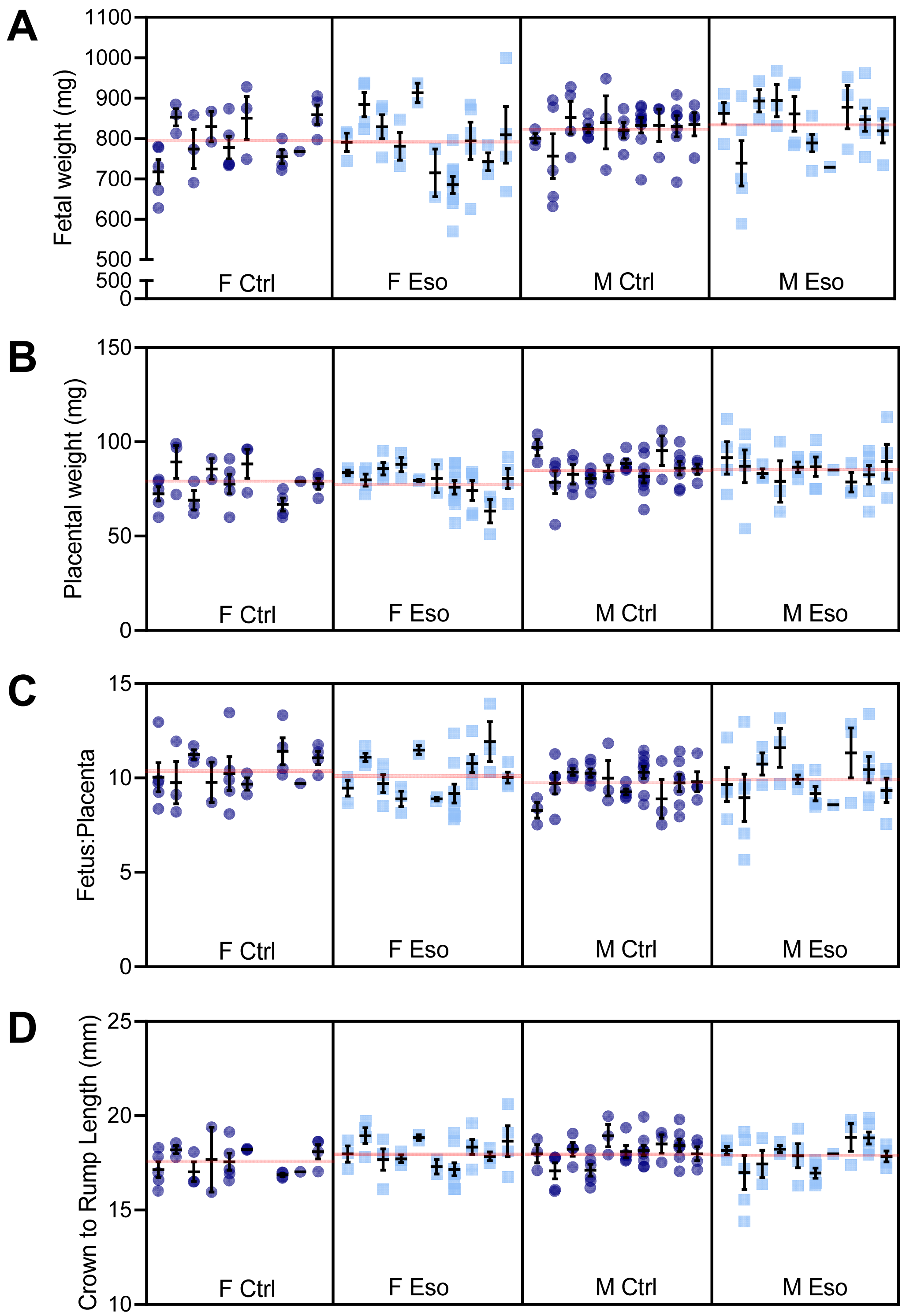

Supplementary Figure S4. Effect of esomeprazole administration to lean L-NAME mice on fetal and placental size split by fetal sex. A) Fetal weight, B) placental weight, C) Ratio to fetal to placental weight, and D) fetal crown-to-rump length. F Ctrl – Female fetuses from control dams, F Eso – female fetuses from dams administered esomeprazole, M Ctrl – male fetuses from

control dams, M Eso – male fetuses from dams administered esomeprazole. Statistical analysis was performed between the fetuses from the control and esomeprazole groups within each sex. Fetal and placental size was not significantly altered in either the female of male fetuses in the esomeprazole group compared to controls. Results are presented from each dam (n=10/group), mean ± SEM. The red line across each box represents the mean of each group.

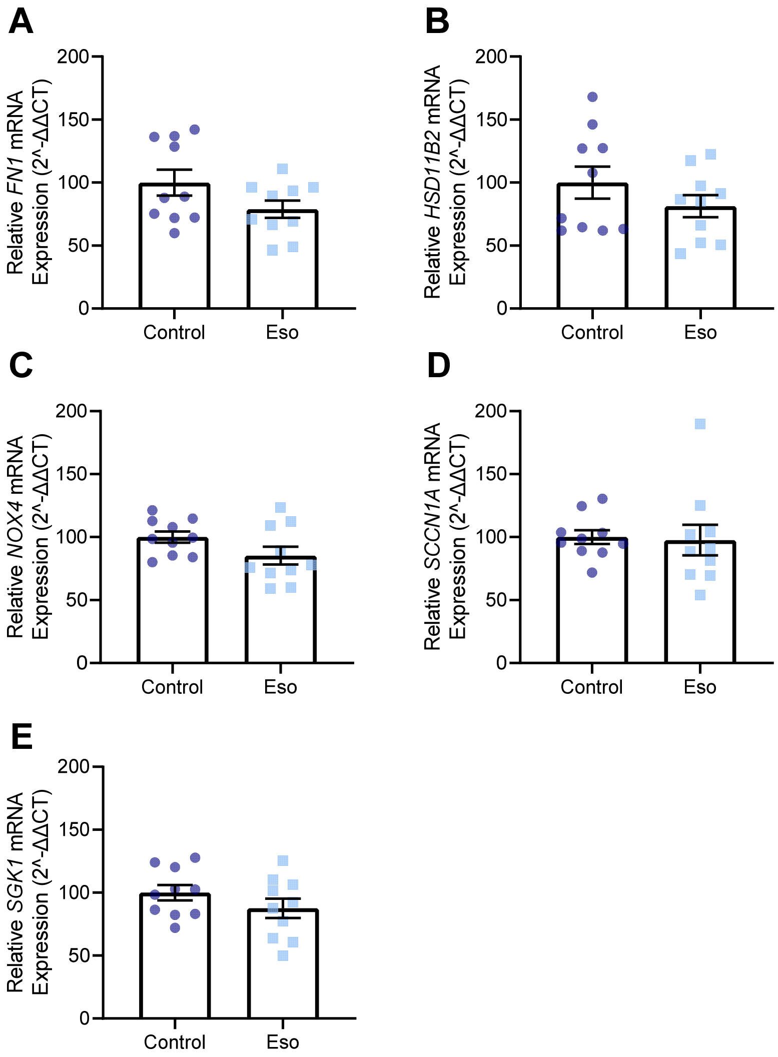

Supplementary Figure S5. Effect of esomeprazole on the expression of genes associated with kidney function in normal pregnant mice receiving L-NAME. Expression of A) Hsd11b2, B) Nox4, C) Fn1, D) Sgk1 and E) Sccn1a (assessed via qPCR) was not significantly different in kidney from mice administered L-NAME with esomeprazole, compared to kidneys from control (no esomeprazole) mice. Data presented as mean ± SEM. n = 10 mice/group.

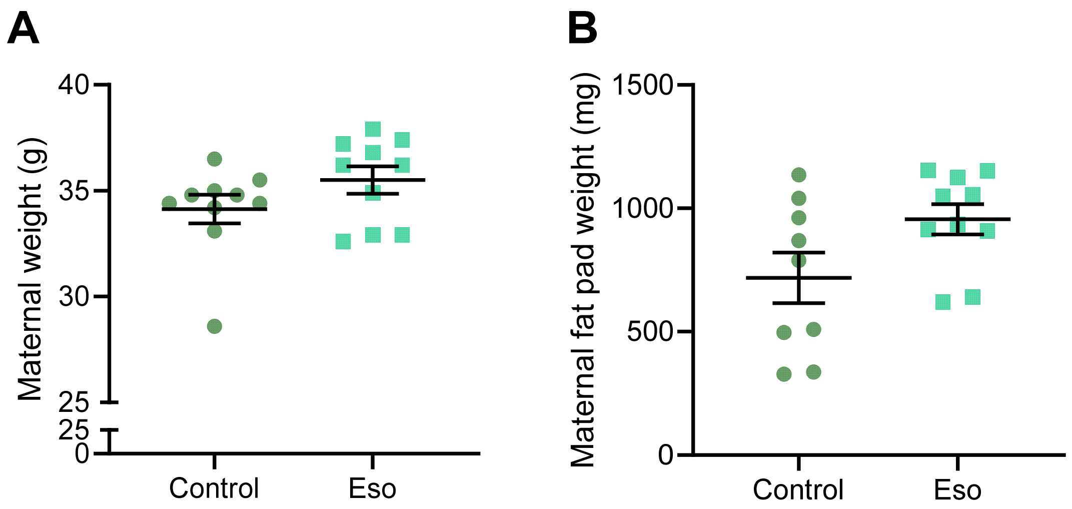

Supplementary Figure S6. Esomeprazole effect on maternal body and fat pad weight in obese L-NAME mice. Maternal weight and fat pad weight were measured at E17.5 of pregnancy. Esomeprazole treatment did not significantly alter the maternal body or fat pad weight of obese L-NAME mice compared to treatment with the vehicle control. Data presented as mean ± SEM; n=10 mice/group. Fat pad weight data missing for n=1 control mouse.

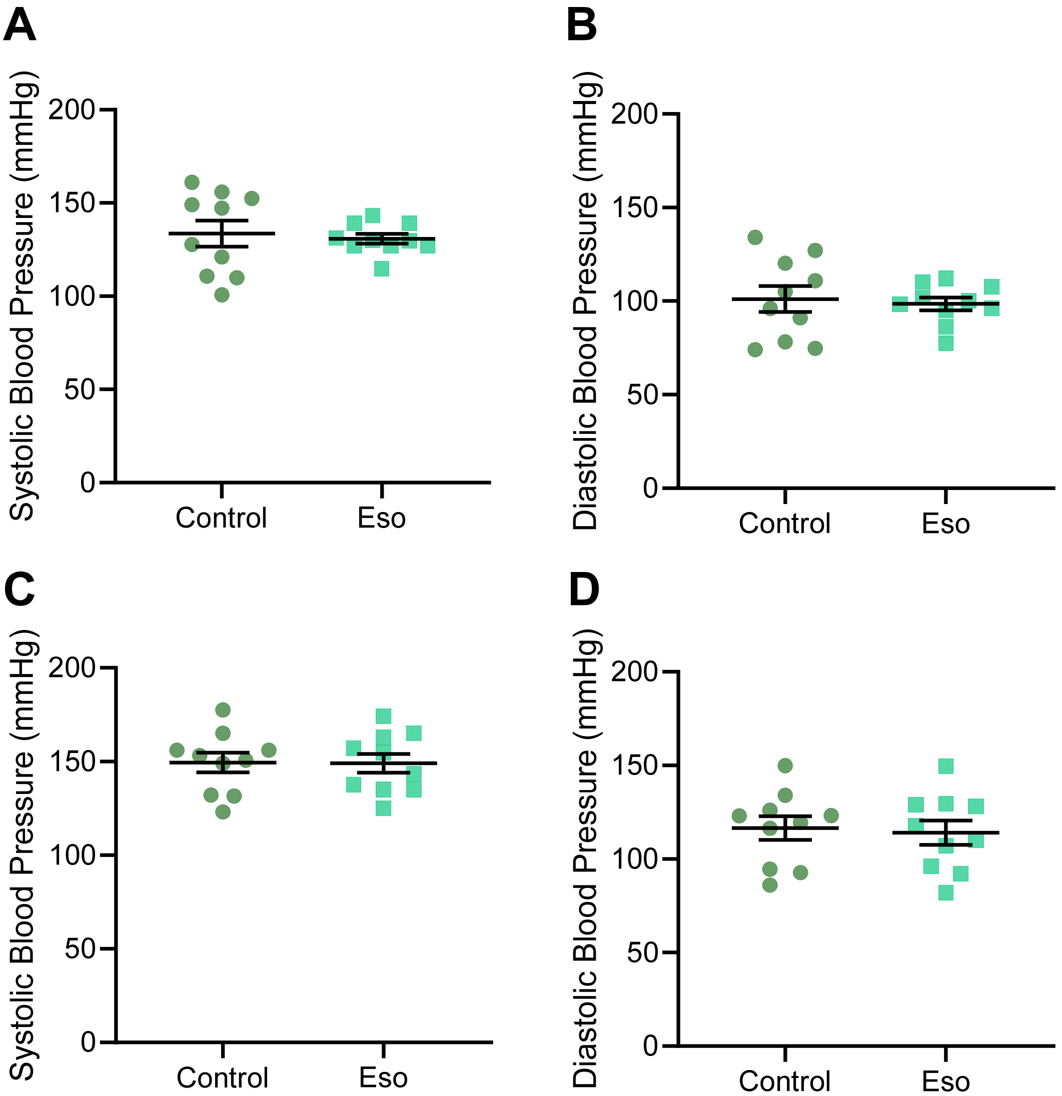

Supplementary Figure S7. Systolic and diastolic blood pressure of obese L-NAME mice treated with esomeprazole. Blood pressures were measured at E14.5 (A, B) and E17.5 (C, D) of pregnancy via tail cuff plethysmography. Esomeprazole treatment did not alter systolic or diastolic blood pressure at either time point from control levels. Data presented as mean ± SEM; n=10 mice/group.

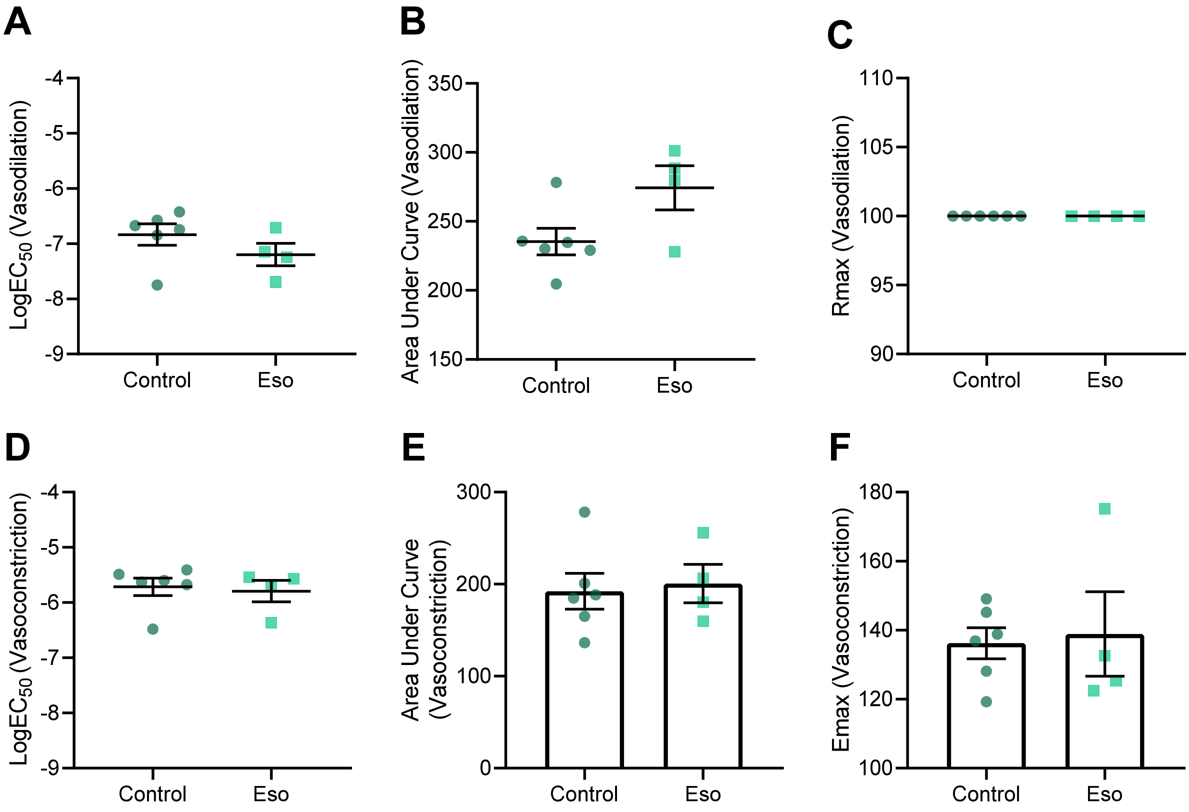

Supplementary Figure S8. Effect of esomeprazole on vascular reactivity of mesenteric arteries collected from obese L-NAME mice – corresponding to myograph curves generated in Figure 7. A) Vasodilation LogEC50, B) vasodilation area under the curve, and C) maximum relaxation (Rmax) to acetylcholine. D) Vasoconstriction LogEC50, E) vasoconstriction area under the curve, and F) maximum constriction (Emax) to phenylephrine. None of the parameters assessed were significantly altered in the arteries collected from the obese L-NAME mice treated with esomeprazole compared to control. Data presented as mean ± SEM; n=4-6 mice/group.

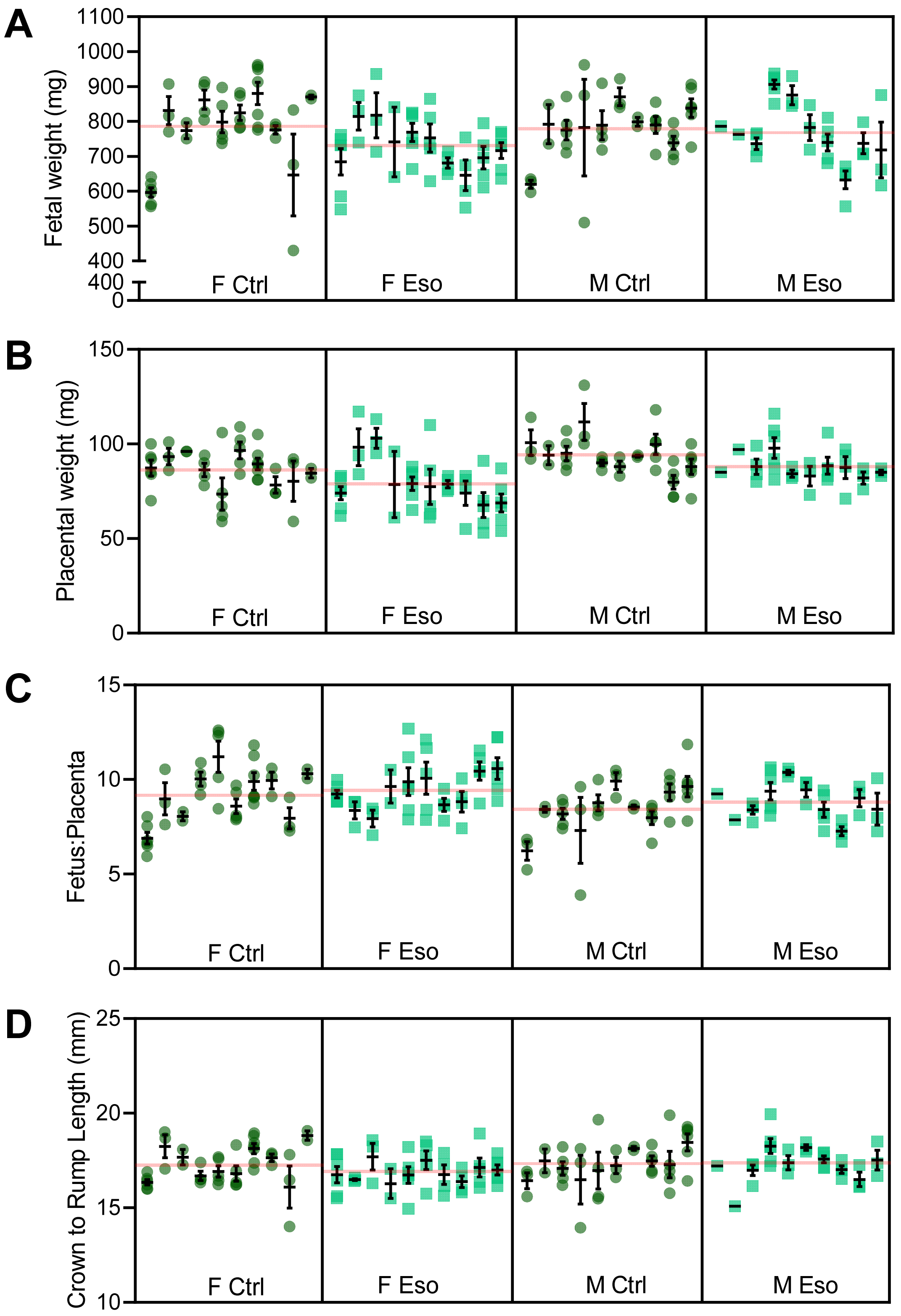

Supplementary Figure S9. Effect of esomeprazole administration to obese L-NAME mice on fetal and placental size split by fetal sex. A) Fetal weight, B) placental weight, C) Ratio to fetal

to placental weight, and D) fetal crown-to-rump length. F Ctrl – Female fetuses from control dams, F Eso – female fetuses from dams administered esomeprazole, M Ctrl – male fetuses from control dams, M Eso – male fetuses from dams administered esomeprazole. Statistical analysis was performed between the fetuses from the control and esomeprazole groups within each sex. Fetal and placental size was not significantly altered in either the female of male fetuses in the esomeprazole group compared to controls. Results are presented from each dam (n=10/group), mean ± SEM. The red line across each box represents the mean of each group.

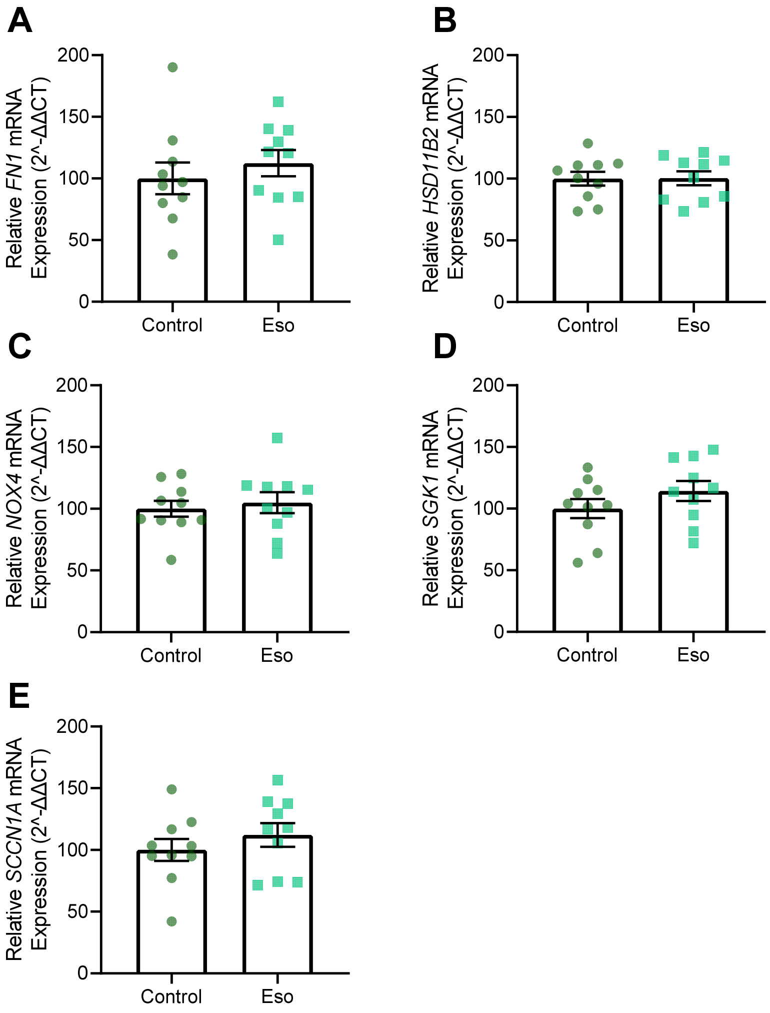

Supplementary Figure S10. Effect of esomeprazole on the expression of genes involved in kidney function in obese pregnant mice receiving L-NAME. Expression of A) FN1, B) Hsd11b2, C) Nox4, D) Sgk1 and E) Sccn1a (assessed via qPCR) were not significantly altered in the kidney of obese L-NAME pregnant dams treated with esomeprazole compared to control. Data presented as mean ± SEM; n = 10 mice/group.
